# Clinical risk stratification model for advanced colorectal neoplasia in persons with negative fecal immunochemical test results

**DOI:** 10.1371/journal.pone.0191125

**Published:** 2018-01-11

**Authors:** Yoon Suk Jung, Chan Hyuk Park, Nam Hee Kim, Jung Ho Park, Dong Il Park, Chong Il Sohn

**Affiliations:** 1 Division of Gastroenterology, Department of Internal Medicine, Kangbuk Samsung Hospital, Sungkyunkwan University School of Medicine, Seoul, Korea; 2 Department of Internal Medicine, Hanyang University Guri Hospital, Hanyang University College of Medicine, Guri, Korea; 3 Preventive HealthCare, Kangbuk Samsung Hospital, Sungkyunkwan University School of Medicine, Seoul, Korea; The University of Tokyo, JAPAN

## Abstract

**Objectives:**

The fecal immunochemical test (FIT) has low sensitivity for detecting advanced colorectal neoplasia (ACRN); thus, a considerable portion of FIT-negative persons may have ACRN. We aimed to develop a risk-scoring model for predicting ACRN in FIT-negative persons.

**Materials and methods:**

We reviewed the records of participants aged ≥40 years who underwent a colonoscopy and FIT during a health check-up. We developed a risk-scoring model for predicting ACRN in FIT-negative persons.

**Results:**

Of 11,873 FIT-negative participants, 255 (2.1%) had ACRN. On the basis of the multivariable logistic regression model, point scores were assigned as follows among FIT-negative persons: age (per year from 40 years old), 1 point; current smoker, 10 points; overweight, 5 points; obese, 7 points; hypertension, 6 points; old cerebrovascular attack (CVA), 15 points. Although the proportion of ACRN in FIT-negative persons increased as risk scores increased (from 0.6% in the group with 0–4 points to 8.1% in the group with 35–39 points), it was significantly lower than that in FIT-positive persons (14.9%). However, there was no statistical difference between the proportion of ACRN in FIT-negative persons with ≥40 points and in FIT-positive persons (10.5% vs. 14.9%, *P* = 0.321).

**Conclusions:**

FIT-negative persons may need to undergo screening colonoscopy if they clinically have a high risk of ACRN. The scoring model based on age, smoking habits, overweight or obesity, hypertension, and old CVA may be useful in selecting and prioritizing FIT-negative persons for screening colonoscopy.

## Introduction

Colorectal cancer (CRC) is the third most common cancer in men and the second most common in women, and it is the fourth most common cause of cancer deaths worldwide [1]. Moreover, the CRC incidence is rapidly increasing, especially in Asian countries [2]. However, CRC is one of the preventable cancers. The removal of precursor lesions (adenomatous polyps) or early-stage CRC has been effective in reducing the incidence and mortality of CRC [3]. Therefore, many efforts have been made to detect early-stage CRC or its precursor lesions.

The fecal immunochemical test (FIT) is now recognized as the best currently available noninvasive assessment in CRC screening programs [4,5]. Screening with FITs has been proven an effective method for detecting a large portion of CRC cases in asymptomatic average-risk populations [4]. Previous studies have shown that FIT has high specificity (90–96%) and good sensitivity (73–88%) for detecting CRC [4]. However, in advanced colorectal neoplasia (ACRN), which consists of advanced adenoma and CRC, FIT reportedly has low sensitivity, ranging from 27% to 32% [6–9]. These results suggest that a considerable proportion of persons with ACRN will have a negative FIT result. Given the low sensitivity of FIT for ACRN detection, it may be difficult for physicians to assure that persons with negative results of FIT have no need to undergo screening colonoscopy.

In this situation, a scoring system that stratifies the risk of ACRN in persons with negative FIT results is required to guide clinical practices. Such a scoring system would be helpful in identifying certain persons who need to undergo colonoscopy despite having a negative FIT result. In addition, such a scoring system can be used in selecting and prioritizing FIT-negative persons for screening colonoscopy. In this study, therefore, we aimed to develop a clinical risk stratification scoring system for predicting the risk of ACRN among persons with negative FIT results.

## Methods

The Kangbuk Samsung Health Study is a cohort study of South Korean men and women aged 18 years or older who underwent a comprehensive annual or biennial health examination at the clinics of the Kangbuk Samsung Hospital Total Healthcare Center in Seoul and Suwon, South Korea. The study population consisted of a subset of Kangbuk Samsung Health Study participants who had undergone colonoscopy and FIT as part of a comprehensive health examination from 2010 to 2014. We retrospectively analyzed data obtained from a prospectively established cohort. The setting of the study was a health examination center, not a clinic. Before colonoscopy, the participants were interviewed by general practitioners to ensure that they were asymptomatic (i.e., they had no abdominal pain or hematochezia). Participants with any gastrointestinal symptoms were referred for appropriate diagnostic testing and treatment.

The participants were limited to persons 40 years old or older. The exclusion criteria were as follows: (i) previous colonic examination, colorectal surgery, or colorectal neoplasia (CRN); (ii) a history of inflammatory bowel disease; (iii) ischemic or infectious colitis diagnosed during colonoscopy; (iv) poor bowel preparation; and (v) incomplete data for analysis. Poor bowel preparation was defined as “large amounts of solid fecal matter found, precluding a satisfactory study; unacceptable preparation; and <90% visible mucosa” [10].

This study was approved by the institutional review board of Kangbuk Samsung Hospital, which exempted the requirement for informed consent because we accessed only de-identified data, retrospectively.

### Measurements

We recorded age, sex, height, weight, family history of CRC, smoking habits, drug history, and comorbidities including hypertension, diabetes, dyslipidemia, old cerebrovascular attack (CVA), and fatty liver, by using our electronic medical database. Data on family history of CRC, smoking habits, and drug history were collected by using a self-administered questionnaire before colonoscopy. Smoking habits were defined by using the criteria of the National Health Interview Survey, as follows: current smoker, former smoker, and never smoker [11]. Body mass index (BMI) was classified into the following 3 groups according to cut-off values for an Asian population: <23 kg/m^2^, 23–27 kg/m^2^, and ≥27 kg/m^2^ [12–14]. Fatty liver was evaluated by means of transabdominal ultrasonography. In addition, we reviewed the colonoscopic findings and results of histopathologic examination to identify participants who had ACRN.

To evaluate the risk of ACRN in FIT-negative persons, we identified factors associated with ACRN in FIT-negative participants. Then, we developed a risk-scoring model for predicting ACRN based on the associated factors.

### Colonoscopy and histopathologic examination

All participants were instructed to discontinue antiplatelet agents for 7 days and anticoagulants for 5 days, with the permission of the physician who prescribed the medication. All colonoscopies were performed by experienced, board-certified endoscopists, by using an Evis Lucera^™^ CV-260 colonoscope (Olympus Medical Systems, Tokyo, Japan). The bowels were cleansed with 4 L polyethylene glycol solution. Suspicious neoplastic lesions were examined through biopsy, or removed with polypectomy or endoscopic mucosal resection.

All specimens obtained from biopsy, polypectomy, or endoscopic mucosal resection were evaluated through histopathologic examination by experienced gastrointestinal pathologists. CRN was defined as a cancer or adenoma. ACRN was defined as a cancer or advanced adenoma. Advanced adenoma was defined as the presence of one of the following features: diameter ≥10 mm, tubulovillous or villous structure, and high-grade dysplasia [15].

### Fecal immunochemical test

All participants collected a one-time stool sample at home, using a sampling tube (Eiken Chemical Company, Tokyo, Japan) containing 2.0 mL buffer designed to minimize hemoglobin degradation, within 3 days before initiating bowel cleansing for colonoscopy.

The collected fecal material was sent to the laboratory sealed in a plastic bag. Fecal hemoglobin quantitation was performed by using OC-SENSOR DIANA (Eiken Chemical Company). FIT results were expressed in nanograms of hemoglobin per milliliter of buffer (ng Hb/mL), and the FIT positivity cutoff value was set at 100 ng Hb/mL (equivalent to 20 μg Hb/g feces) [16].

### Statistical analysis

Continuous variables such as age are presented as mean with standard deviation, and they were compared by using Student’s t-test. Categorical variables are presented as numbers with percentages. To identify factors associated with ACRN, univariable logistic regression analysis was performed. Age, sex, smoking habits, BMI, use of nonsteroidal anti-inflammatory drugs (NSAIDs), and variables that were significant in the univariable analysis were included in the multivariable logistic regression models. Calibration was evaluated with the Hosmer-Lemeshow goodness-of-fit test.

Next, we assigned point scores to each significant variable in the model by dividing the coefficient of regression for significant predictors by the smallest coefficient (assigning a score of 1 for the predictor with the smallest coefficient) [17,18]. FIT-negative participants were classified according to the risk scores, as follows: 0–4, 5–9, 10–14, 15–19, 20–24, 25–29, 30–34, 35–39, and ≥40. Finally, the proportion of ACRN in each risk group was compared with that in FIT-positive participants by using the chi-square test. A *P*-value of <0.05 was considered significant for group comparisons. All statistical analyses were conducted with the statistical software R (version 3.2.3; R Foundation for Statistical Computing, Vienna, Austria).

## Results

### Participants and baseline characteristics

We reviewed the medical records of 17,907 participants ≥40 years old who had undergone both colonoscopy and FIT. Of these, 1,411 were excluded because a history of colonic examination, colorectal surgery, or CRN. In addition, 45 participants with inflammatory bowel disease were excluded. Thirteen participants who were diagnosed as having ischemic or infectious colitis during the current colonoscopy were excluded because such conditions could influence the FIT results. Of the remaining 16,438 participants, 1,533 were excluded because of poor bowel preparation and 2,635 were excluded because of incomplete data. Ultimately, 12,270 participants were included in the study.

The baseline characteristics of the participants and the prevalence of colorectal neoplastic lesions are shown in Table 1. The number of participants with positive results of FIT was 397 (3.2%). The mean age was higher in the FIT-positive group than in the FIT-negative group (47.5 ± 7.4 vs. 46.7 ± 6.8 years, *P* = 0.041). The proportion of male participants was 71.3% and 71.2% in the FIT-positive and FIT-negative groups, respectively. Smoking habits; obesity; family history of CRC; comorbidities including hypertension, diabetes, dyslipidemia, old CVA, and fatty liver; and use of NSAIDs did not differ between the groups. The prevalences of ACRN and overall CRN were 14.9% and 34.3%, respectively, in the FIT-positive group, whereas they were 2.1% and 21.6%, respectively, in the FIT-negative group.

**Table 1:** Baseline characteristics according to the results of the fecal immunochemical test.

Variable	FIT (+)	FIT (-)	*P*-value
N	397	11873	
Age, mean±SD, year	47.5±7.4	46.7±6.8	0.041
Male, n (%)	283 (71.3)	8452 (71.2)	0.966
Smoking habit, n (%)			0.193
Never smoker	175 (44.1)	5783 (48.7)	
Former smoker	116 (29.2)	3172 (26.7)	
Current smoker	106 (26.7)	2918 (26.7)	
Body mass index (kg/m^2^)			0.494
<23	137 (34.5)	4292 (36.1)	
23–27	190 (47.9)	5739 (48.3)	
≥27	70 (17.6)	1842 (15.5)	
Family history of CRC, n (%)	19 (4.8)	509 (4.3)	0.630
Hypertension, n (%)	35 (8.8)	1067 (9.0)	0.907
Diabetes, n (%)	34 (8.6)	840 (7.1)	0.256
Old cerebrovascular attack, n (%)	2 (0.5)	60 (0.5)	0.997
Dyslipidemia, n (%)	16 (4.0)	599 (5.0)	0.362
Fatty liver, n (%)	150 (37.8)	4428 (37.3)	0.843
Use of NSAIDs, n (%)	21 (5.3)	526 (4.4)	0.414
CRN, n (%)	136 (34.3)	2559 (21.6)	<0.001
ACRN, n (%)	59 (14.9)	255 (2.1)	<0.001
Advanced adenoma	50 (12.6)	251 (2.1)	<0.001
Proximal colon	13 (3.3)	98 (0.8)	0.218^a^
Distal colon	35 (8.8)	145 (1.2)	
Both	2 (0.5)	8 (0.07)	
Cancer	9 (2.3)	4 (0.03)	<0.001
Proximal colon	2 (0.5)	1 (0.008)	0.234^a^
Distal colon	5 (1.3)	3 (0.03)	
Both	2 (0.5)	0 (0.0)	

^a^These *P*-values represent the statistical difference of advanced adenoma or cancer according to the tumor location (proximal colon vs. distal colon vs. both) and FIT results.

FIT, fecal immunochemical test; BMI, body mass index; CRC, colorectal cancer; NSAID, nonsteroidal anti-inflammatory drug; CRN, colorectal neoplasia; ACRN, advanced colorectal neoplasia; SD, standard deviation

### Logistic regression model for predicting ACRN

The univariable and multivariable logistic regression models for the prediction of ACRN are shown in Table 2. In the FIT-positive group, only age was an independent factor for ACRN. In the FIT-negative group, age, sex, smoking habits, BMI, hypertension, and old CVA were associated with the risk of ACRN in the univariable analysis. In the multivariable logistic regression model, age, current smoking, being overweight (BMI 23–27 kg/m^2^) or obese (BMI >27 kg/m^2^), hypertension, and old CVA were independent risk factors for ACRN in FIT-negative participants. Our predictive model showed good calibration with the goodness-of-fit test (*P* = 0.930).

**Table 2:** Factors associated with advanced colorectal neoplasia according to the fecal immunochemical test results.

Variable	Univariable analysis				Multivariable analysis					
	FIT(+) individuals (n = 397)		FIT(-) individuals (n = 11873)		FIT(+) individuals (n = 397)			FIT(-) individuals (n = 11873)		
	OR (95% CI)	*P*-value	OR (95% CI)	*P*-value	Coefficient	OR (95% CI)	*P*-value	Coefficient	OR (95% CI)	*P*-value
Age	1.08 (1.04–1.12)	<0.001	1.06 (1.05–1.08)	<0.001	0.078	1.08 (1.04–1.12)	<0.001	0.066	1.07 (1.05–1.08)	<0.001
Male	2.18 (1.10–4.71)	0.034	1.48 (1.11–2.02)	0.010	0.729	2.07 (0.84–5.30)	0.117	0.181	1.20 (0.81–1.77)	0.362
Smoking habit										
Never smoker	1		1		0.000	1		0.000	1	
Former smoker	1.71 (0.87–3.39)	0.120	1.45 (1.06–1.96)	0.019	0.114	1.12 (0.50–2.59)	0.786	0.271	1.31 (0.91–1.91)	0.151
Current smoker	1.91 (0.96–3.80)	0.063	1.86 (1.38–2.50)	<0.001	0.303	1.35 (0.61–3.09)	0.464	0.666	1.95 (1.36–2.80)	<0.001
BMI, kg/m^2^	0.98 (0.54–1.73)	0.941								
<23	1		1		0.000	1		0.000	1	
23–27	1.12 (0.60–2.12)	0.725	1.64 (1.22–2.22)	0.001	0.004	1.00 (0.52–1.99)	0.990	0.336	1.40 (1.04–1.91)	0.030
≥27	1.16 (0.50–2.56)	0.721	1.78 (1.22–2.58)	0.003	0.108	1.11 (0.46–2.59)	0.804	0.438	1.55 (1.05–2.26)	0.024
Family history of CRC	2.14 (0.67–5.86)	0.159	1.20 (0.65–2.03)	0.519	0.616	1.85 (0.54–5.39)	0.283	0.213	1.24 (0.67–2.10)	0.465
Hypertension	1.21 (0.44–2.86)	0.691	1.74 (1.21–2.45)	0.002				0.392	1.48 (1.02–2.09)	0.032
Diabetes	2.25 (0.95–4.96)	0.052	1.44 (0.93–2.15)	0.088						
Dyslipidemia	0.81 (0.13–3.01)	0.787	1.18 (0.67–1.93)	0.537						
Old cerebrovascular attack	5.81 (0.23–148.31)	0.216	4.20 (1.46–9.62)	0.002				0.992	2.70 (0.91–6.37)	0.041
Fatty liver	1.06 (0.60–1.86)	0.837	1.22 (0.95–1.57)	0.120						
Use of NSAIDs	0.95 (0.22–2.93)	0.939	1.16 (0.63–1.96)	0.601	-0.095	0.91 (0.19–3.16)	0.892	<0.001	1.00 (0.54–1.71)	0.998

FIT, fecal immunochemical test; BMI, body mass index; CRC, colorectal cancer; NSAID, nonsteroidal anti-inflammatory drug; OR, odds ratio; CI, confidence interval

On the basis of the logistic regression analysis, point scores were assigned as follows among FIT-negative participants: (i) age (per year from 40 years old), 1 point; (ii) current smoker, 10 points; (iii) BMI <23 kg/m^2^, 0 point/BMI 23–27 kg/m^2^, 5 points/BMI ≥27 kg/m^2^, 7 points; (iv) hypertension, 6 points; and (v) old CVA, 15 points (Table 3).

**Table 3:** Point assignments for predicting advanced colorectal neoplasia in persons with negative fecal immunochemical test results.

Risk factor	Points
Age, /year from 40 years old	1
Current smoker	10
BMI, kg/m^2^	
<23	0
23–27 (overweight)	5
≥27 (obese)	7
Hypertension	6
Old cerebrovascular attack	15

BMI, body mass index

### Distribution of risk scores and proportion of ACRN

**Fig 1:**
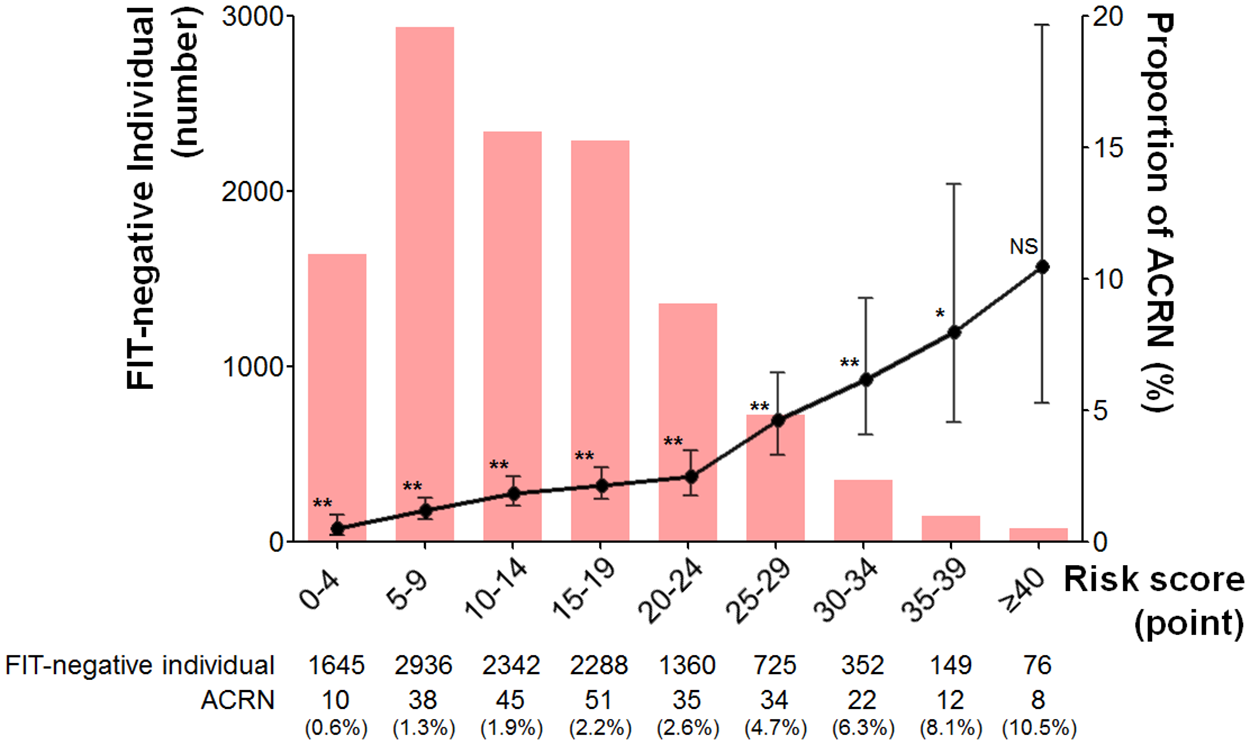
The number of persons and proportion of ACRN according to the risk scores in the FIT-negative group. Red bar graphs represent the number of persons in each risk group. Black points and bars represent the proportions of ACRN and their 95% confidence intervals. Statistical significance represents the difference in proportion of ACRN between each risk group and the FIT-positive group. ACRN, advanced colorectal neoplasia; FIT, fecal immunochemical test; NS, not significant. **P < 0.01, *P < 0.05.

The distribution of ACRN risk scores in FIT-negative participants is shown in Fig 1. Most participants belonged to the ≤39 points group (11,797 participants, 99.4%). Although the proportion of ACRN increased as risk scores increased in FIT-negative participants (from 0.6% in the group with 0–4 points to 8.1% in the group with 35–39 points), it was significantly lower than that in the FIT-positive group (14.9%). However, there was no statistical difference between the proportion with ACRN in FIT-negative persons with ≥40 points and in FIT-positive persons (10.5% vs. 14.9%, *P* = 0.321). Using the cut-off value of 40 points, the sensitivity and specificity of the model for discriminating ACRN were 3.1% and 99.4%, respectively.

### Risk of CRN in FIT-negative participants

The risk factors for overall CRN were evaluated by using the logistic regression model, as shown in S1 Table. In the FIT-negative group, age, male sex, current smoker, overweight or obesity, family history of CRC, hypertension, diabetes, and fatty liver were identified as independent risk factors for CRN. Assigned point scores for the risk of CRN in FIT-negative participants are shown in S2 Table. Additionally, the distribution of CRN risk scores in FIT-negative participants is shown in S1 Fig. The FIT-negative group with 35–39 and ≥40 points had a higher risk of CRN than the FIT-positive group (vs. FIT-positive group: 35–39 points group, 41.6% [95% CI, 37.0–46.2%], *P* = 0.030; ≥40 points group, 51.8% [46.5–57.0%], *P* < 0.001). Using a cut-off value of 30 points, the sensitivity and specificity of the model for discriminating CRN were 27.0% and 89.0%, respectively.

## Discussion

Many countries have adopted FIT in their population-based CRC screening program because FIT is effective for detecting CRC and is noninvasive [4,5]. However, considering that FIT has low sensitivity for detecting ACRN (27–32%) [6–9], a nonnegligible portion of persons with negative results of FIT may have ACRN. To identify these persons in whom ACRN diagnosed through FIT-based screening might be missed, we developed a clinical risk stratification scoring system for predicting the risk of ACRN among persons with negative FIT results. In our study, age, smoking, obesity, hypertension, and old CVA were independent risk factors for ACRN in FIT-negative participants. On the basis of the results, point scores were assigned as follows among FIT-negative participants: (i) age (per year from 40 years old), 1 point; (ii) current smoker, 10 points; (iii) BMI <23 kg/m^2^, 0 point/BMI 23–27 kg/m^2^, 5 points/BMI ≥27 kg/m^2^, 7 points; (iv) hypertension, 6 points; and (v) old CVA, 15 points. Although the risk of ACRN in FIT-negative persons increased as risk scores increased, it was lower than that in FIT-positive persons. However, the proportion of ACRN in the FIT negative group with ≥40 points was not significantly different from that in the FIT positive group (9.8% vs. 14.9%, *P* = 0.321). Although FIT is a much stronger predictor of ACRN than clinical risk factors, even FIT-negative persons may undergo colonoscopy if they clinically have a high risk of ACRN. In other words, persons with a risk score of ≥40 points may be recommended to undergo primary screening with colonoscopy rather than FIT before colonoscopy.

Currently, several models stratify the risk of ACRN. However, there have been no scoring models focused on FIT-negative persons. All the currently available ACRN risk-stratification models, such as the Asia-Pacific Colorectal Screening score, the Korean Colorectal Screening (KCS) score, and the risk prediction index in the United States, include age and smoking habits because they are important risk factors for ACRN [19–21]. The KCS scoring model also includes BMI because obesity is closely related to the risk of ACRN [20]. Similar to the existing models, our scoring model for predicting ACRN in FIT-negative persons included age, smoking habits, and obesity. Our model also included hypertension and old CVA. Interestingly, old CVA was a significant risk factor for ACRN, and it was assigned the highest score in FIT-negative persons.

To date, only a few studies have reported the relationship between CVA and CRC. A Danish population-based study showed that patients with stroke were at 42% increased risk of having a CRC diagnosis within the first year of stroke diagnosis [22]. In another Japanese study, women with a medical history of stroke had increased risk of rectal cancer, although it did not reach statistical significance (hazard ratio, 2.99; 95% CI, 0.72–12.45) [23]. The development of these two diseases seems to involve chronic inflammation, which is known to be crucial in the atherosclerotic process [24] and is also important in CRC carcinogenesis [25]. Stroke and CRN also share some risk factors such as smoking, obesity, and metabolic syndrome, all of which have been associated with systemic chronic inflammation [26]. Atherosclerosis may be a potent risk factor for ACRN in FIT-negative persons.

The clinical application of our model is as follows. The risk score exceeds 40 points if a person is 60 years or older (≥20 points), current smoker (10 points), overweight or obese (5 or 7 points), and has hypertension (6 points). Old and overweight (or obese) smokers with hypertension should be strongly recommended to undergo colonoscopy even if they have negative FIT results. As another example, the risk score exceeds 40 points if a person is ≥60 years old (≥20 points) and has a history of both stroke (15 points) and hypertension (6 points). Old persons (≥60 years) with a history of stroke and hypertension should also undergo colonoscopy even if they have negative FIT results. In other words, these persons may be recommended to undergo primary screening with colonoscopy rather than FIT. In addition, persons with 30–34 points or 35–39 points had considerable risk of ACRN (6.3% and 8.1%, respectively). Although we did not recommend primary colonoscopy in these persons because of cost-effectiveness, we should consider that they have a relatively high risk of ACRN, even with negative results on FIT.

Contrary to FIT-negative persons, in FIT-positive persons, only age was associated with the risk of ACRN. These results indicate that FIT-positive persons should undergo colonoscopy regardless of clinical risk factors such as smoking habit, obesity, and underlying diseases.

Meanwhile, the model for predicting CRN included sex, family history of CRC, diabetes, and fatty liver, as well as age, smoking habit, obesity, and hypertension. Unlike the results of ACRN, FIT-negative persons with ≥35 points rather had a higher risk of CRN than FIT-positive persons. This finding implies that the results of FIT can well reflect the presence of ACRN, rather than CRN. In terms of predicting CRN, clinical risk stratification may be more effective than FIT.

To the best of our knowledge, this is the first study to develop a risk-scoring model for predicting ACRN and CRN among FIT-negative persons. Nonetheless, it had several limitations. First, our study was not population based and our cohort was recruited from two medical examination centers in Korea. Therefore, there was likely some degree of selection bias. Second, our cohort only included 13 patients with CRC. Therefore, we analyzed the risk factor associated with ACRN rather than CRC. Risk factors for CRC in FIT-negative individuals could not be clarified through the current study. However, we believe that understanding of risk factors for ACRN is also important because detection and removal of advanced adenoma can help prevent development of CRC. Third, our cohort consisted of low risk population for CRC. The mean age was less than 50 years and a FIT-positivity rate was about 3%. Although our scoring system included age as an independent risk factor for ACRN, readers should exercise caution when generalizing our findings to other populations. Fourth, we could not divide the cohort into derived and validation cohorts, because the number of individuals with ACRN was relatively small. Although we showed the good calibration of the logistic regression models by the Hosmer-Lemeshow goodness-of-fit test, validation of our scoring model should be performed through another independent population. Fifth, we adopted a one-specimen FIT; thus, the ability of FIT for ACRN detection may have been underestimated. Sixth, there was likely some degree of recall bias. For example, a historical risk factor that can be very remote, such as an old CVA, might not be as accurately registered as more objective or recent factors. Our scoring model based on historical risk factors may have some limitations in clinical application. Finally, although our model could determine the high-risk group among FIT-negative persons, it was not evaluated in terms of cost-effectiveness. Subsequent cost-effective analyses need to be performed to confirm the usefulness of our model.

Despite these limitations, our data provide a better understanding of the risk assessment for ACRN in FIT-negative persons. FIT-negative persons with high risk scores (≥40 points) showed high risk of ACRN. There was no statistical difference between the proportion of ACRN in FIT-negative persons with ≥40 points and in FIT-positive persons. Our study suggests that persons with negative FIT results may need to undergo screening colonoscopy if they clinically have a high risk of ACRN. Our scoring model that includes age, smoking habits, overweight or obesity, hypertension, and old CVA may be useful in selecting and prioritizing FIT-negative persons for screening colonoscopy.

## Supporting information

S1 Fig The number of persons and proportion of overall colorectal neoplasia according to the risk scores in the FIT-negative group. Blue bar graphs represent the number of persons in each risk group. Black points and bars represent the proportions of colorectal neoplasia and their 95% confidence intervals. Statistical significance represents the difference in proportion of CRN between each risk group and the FIT-positive group. CRN, colorectal neoplasia; FIT, fecal immunochemical test; NS, not significant. **P < 0.01, *P < 0.05.
(TIF)

S1 Table Factors associated with overall colorectal neoplasia in persons with negative fecal immunochemical test results. (DOCX)

S2 Table Point assignments for predicting overall colorectal neoplasia in persons with negative fecal immunochemical test results. (DOCX)
