# Supplementary figures and images for: Clinical risk stratification model for advanced colorectal neoplasia in persons with negative fecal immunochemical test results
Source: PLoS One. 2018 Jan 11;13(1):e0191125. doi: 10.1371/journal.pone.0191125 (PMC5764375; doi:10.1371/journal.pone.0191125)

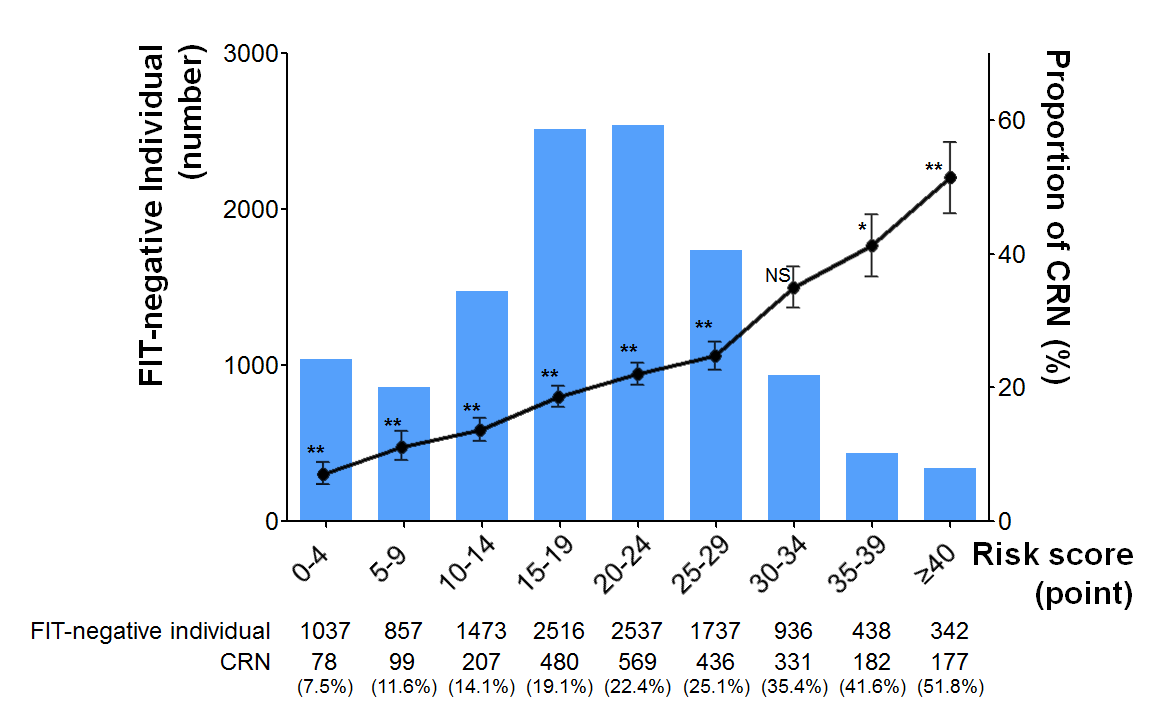

Supplement: S1 Fig — Blue bar graphs represent the number of persons in each risk group. Black points and bars represent the proportions of colorectal neoplasia and their 95% confidence intervals. Statistical significance represents the difference in proportion of CRN between each risk group and the FIT-positive group. CRN, colorectal neoplasia; FIT, fecal immunochemical test; NS, not significant. **P < 0.01, *P < 0.05. (TIF) [file pone.0191125.s001.tif]
