# Supplementary material for: Clinical risk stratification model for advanced colorectal neoplasia in persons with negative fecal immunochemical test results
Source: PLoS One. 2018 Jan 11;13(1):e0191125. doi: 10.1371/journal.pone.0191125 (PMC5764375; doi:10.1371/journal.pone.0191125)
Supplement: S1 Table — (DOCX) [file pone.0191125.s002.docx]

| **S1 Table. Factors associated with overall colorectal neoplasia according to the fecal immunochemical test results.** | | | | | | | | | | | | | | |
| --- | --- | --- | --- | --- | --- | --- | --- | --- | --- | --- | --- | --- | --- | --- |
| Variable | | Univariable analysis | | | |  |  | Multivariable analysis | | | | | | |
|  |  | FIT(+) individuals (n=397) | |  | FIT(-) individuals (n=11873) | |  | FIT(+) individuals (n=397) | | |  | FIT(-) individuals (n=11873) | | |
|  |  | OR (95% CI) | *P*-value |  | OR (95% CI) | *P*-value |  | Coefficient | OR (95% CI) | *P*-value |  | Coefficient | OR (95% CI) | *P*-value |
| Age | | 1.09 (1.06-1.13) | <0.001 |  | 1.05 (1.04-1.06) | <0.001 |  | 0.099 | 1.10 (1.07-1.14) | <0.001 |  | 0.057 | 1.06 (1.05-1.07) | <0.001 |
| Male | | 2.63 (1.59-4.48) | <0.001 |  | 1.99 (1.79-2.22) | <0.001 |  | 1.154 | 3.17 (1.61-6.43) | 0.001 |  | 0.631 | 1.88 (1.64-2.16) | <0.001 |
| Smoking habit | |  |  |  |  |  |  |  |  |  |  |  |  |  |
|  | Never smoker | 1 |  |  | 1 |  |  | 0.000 | 1 |  |  | 0.000 | 1 |  |
|  | Former smoker | 1.58 (0.96-2.61) | 0.070 |  | 1.52 (1.37-1.69) | <0.001 |  | -0.228 | 0.80 (0.42-1.49) | 0.477 |  | 0.098 | 1.10 (0.97-1.25) | 0.127 |
|  | Current smoker | 1.58 (0.95-2.63) | 0.080 |  | 1.77 (1.60-1.97) | <0.001 |  | -0.092 | 0.91 (0.48-1.71) | 0.775 |  | 0.354 | 1.42 (1.26-1.62) | <0.001 |
| BMI, kg/m^2^ | |  |  |  |  |  |  |  |  |  |  |  |  |  |
|  | <23 | 1 |  |  | 1 |  |  | 0.000 | 1 |  |  | 0.000 | 1 |  |
|  | 23-27 | 1.66 (1.03-2.72) | 0.040 |  | 1.46 (1.32-1.61) | <0.001 |  | 0.360 | 1.43 (0.84-2.48) | 0.193 |  | 0.136 | 1.15 (1.03-1.28) | 0.014 |
|  | ≥27 | 2.45 (1.34-4.52) | 0.004 |  | 1.69 (1.48-1.93) | <0.001 |  | 0.889 | 2.43 (1.25-4.78) | 0.009 |  | 0.248 | 1.28 (1.11-1.49) | <0.001 |
| Family history of CRC | | 1.13 (0.41-2.87) | 0.808 |  | 1.29 (1.05-1.58) | 0.014 |  | 0.011 | 1.01 (0.33-2.80) | 0.983 |  | 0.306 | 1.36 (1.10-1.67) | 0.004 |
| Hypertension | | 2.19 (1.09-4.43) | 0.028 |  | 1.76 (1.53-2.01) | <0.001 |  | 0.529 | 1.70 (0.77-3.73) | 0.186 |  | 0.448 | 1.56 (1.36-1.80) | <0.001 |
| Diabetes | | 1.58 (0.76-3.21) | 0.208 |  | 1.73 (1.49-2.02) | <0.001 |  |  |  |  |  | 0.202 | 1.22 (1.04-1.44) | 0.015 |
| Dyslipidemia | | 1.98 (0.71-5.49) | 0.183 |  | 1.41 (1.17-1.69) | <0.001 |  |  |  |  |  | 0.012 | 1.01 (0.83-1.23) | 0.904 |
| Old cerebrovascular attack | | 1.93 (0.08-48.96) | 0.644 |  | 1.56 (0.88-2.68) | 0.114 |  |  |  |  |  |  |  |  |
| Fatty liver | | 1.13 (0.74-1.73) | 0.569 |  | 1.42 (1.30-1.56) | <0.001 |  |  |  |  |  | 0.124 | 1.13 (1.02-1.25) | 0.017 |
| Use of NSAIDs | | 0.76 (0.26-1.91) | 0.574 |  | 1.00 (0.80-1.23) | 0.968 |  | -0.413 | 0.66 (0.21-1.86) | 0.451 |  | -0.060 | 0.94 (0.75-1.17) | 0.596 |
| FIT, fecal immunochemical test; BMI, body mass index; CRC, colorectal cancer; NSAID, nonsteroidal anti-inflammatory drug; OR, odds ratio; CI, confidence interval | | | | | | | | | | | | | | |
