# Supplementary material for: Clinical risk stratification model for advanced colorectal neoplasia in persons with negative fecal immunochemical test results
Source: PLoS One. 2018 Jan 11;13(1):e0191125. doi: 10.1371/journal.pone.0191125 (PMC5764375; doi:10.1371/journal.pone.0191125)
Supplement: S2 Table — (DOCX) [file pone.0191125.s003.docx]

| **S2 Table. Point assignments for predicting overall colorectal neoplasia in persons with negative fecal immunochemical test results.** | | |
| --- | --- | --- |
| Risk factor | | Points |
| Age, /year from 40 years old | | 1 |
| Male | | 11 |
| Current smoker | | 6 |
| BMI, kg/m^2^ | |  |
|  | <23 | 0 |
|  | 23-27 (overweight) | 2 |
|  | ≥27 (obese) | 4 |
| Family history of CRC | | 5 |
| Hypertension | | 8 |
| Diabetes | | 4 |
| Fatty liver | | 2 |
| BMI, body mass index; CRC, colorectal cancer | | |
